# Identification of Class I HLA T Cell Control Epitopes for West Nile Virus

**DOI:** 10.1371/journal.pone.0066298

**Published:** 2013-06-10

**Authors:** Saghar Kaabinejadian, Paolo A. Piazza, Curtis P. McMurtrey, Stephen R. Vernon, Steven J. Cate, Wilfried Bardet, Fredda B. Schafer, Kenneth W. Jackson, Diana M. Campbell, Rico Buchli, Charles R. Rinaldo, William H. Hildebrand

**Affiliations:** 1 Department of Microbiology and Immunology, University of Oklahoma Health Sciences Center, Oklahoma City, Oklahoma, United States of America; 2 Department of Infectious Diseases and Microbiology, University of Pittsburgh Graduate School of Public Health, Pittsburgh, Pennsylvania, United States of America; 3 Department of Pathology, University of Pittsburgh, Pittsburgh, Pennsylvania, United States of America; 4 Pure Protein L.L.C., Oklahoma City, Oklahoma, United States of America; University of Texas Medical Branch, United States of America

## Abstract

The recent West Nile virus (WNV) outbreak in the United States underscores the importance of understanding human immune responses to this pathogen. Via the presentation of viral peptide ligands at the cell surface, class I HLA mediate the T cell recognition and killing of WNV infected cells. At this time, there are two key unknowns in regards to understanding protective T cell immunity: 1) the number of viral ligands presented by the HLA of infected cells, and 2) the distribution of T cell responses to these available HLA/viral complexes. Here, comparative mass spectroscopy was applied to determine the number of WNV peptides presented by the HLA-A*11:01 of infected cells after which T cell responses to these HLA/WNV complexes were assessed. Six viral peptides derived from capsid, NS3, NS4b, and NS5 were presented. When T cells from infected individuals were tested for reactivity to these six viral ligands, polyfunctional T cells were focused on the GTL9 WNV capsid peptide, ligands from NS3, NS4b, and NS5 were less immunogenic, and two ligands were largely inert, demonstrating that class I HLA reduce the WNV polyprotein to a handful of immune targets and that polyfunctional T cells recognize infections by zeroing in on particular HLA/WNV epitopes. Such dominant HLA/peptide epitopes are poised to drive the development of WNV vaccines that elicit protective T cells as well as providing key antigens for immunoassays that establish correlates of viral immunity.

## Introduction

West Nile virus (WNV) is a flavivirus that infects avian and mammalian species, including humans [1]. Symptomatic human infections exhibit a severe fever and, in some cases, encephalitis leading to death. Since 1999, more than 30,000 individuals in the United States have become ill with West Nile virus, and in 2012 forty-eight states have reported a total of 5,387 cases of West Nile virus disease in people, including 243 deaths [2]. This is the highest number of West Nile virus disease cases reported in the United States since 2003, with an unusually high percentage (51%) of the reported infections classified as neuroinvasive disease (such as meningitis or encephalitis) [2]. WNV is now endemic in North America where it continues to inflict considerable morbidity and mortality [1], [3].

Historically, adaptive immune mechanisms effectively control WNV so that most infections are asymptomatic [4]–[6]. Humoral responses directed to the lateral ridge of the WNV envelope domain III (DIII) are highly neutralizing while humoral responses to other regions of the envelope, such as the fusion loop of DIII, are less effective at virus neutralization [7], [8]. In instances where antibodies do not prevent viral entry into host cells, CD8^+^ T cells eliminate WNV infected cells. In both humans and in animal models, CD8^+^ T cells clear WNV infected cells from the periphery and central nervous system [9]–[11]. Through the presentation of virus-derived peptide epitopes at the plasma membrane, class I HLA enable CD8^+^ T cell recognition and cytolysis of infected cells. Just as with antibody epitopes, the identification of HLA presented viral peptide epitopes that correspond to protective immunity is of critical importance for T cell vaccine development and for establishing correlates of T cell immunity.

At this time, the number and source of viral ligands revealed to T cells by any given HLA class I molecule has not been tested. Peptide screening data in humans demonstrate that HLA-A, HLA-B and HLA-C present immunogenic WNV peptide ligands to T cells [12], [13], but these screening data do not distinguish HLA/WNV complexes that correlate with protective T cell immunity from those that do not. Preliminary data with HLA-A*02:01 shows that a small number of viral ligands are presented to T cells [13] and that, following infection, T cell responses focus on one dominant envelope epitope SVG9. Other than SVG9, T cell responses to other viral ligands were inconsistent and, for some A2/WNV ligands, undetectable [13]. Therefore, HLA-A2 distills WNV to a handful of ligands for T cell review.

Establishing A2/SVG9 as an immunodominant WNV epitope was key to the development of one WNV vaccine and the testing of another. A Single Chain Trimer DNA plasmid vaccine comprised of HLA-A2 and the immunodominant SVG9 WNV ligand induced robust CD8^+^ T cell responses, enhanced survival, and lowered brain viral burden following a lethal WNV challenge in HLA transgenic mice. The adoptive transfer of these vaccine induced SVG9-specific CD8^+^ T cells further protected mice from an otherwise lethal WNV infections [14]. In humans, vaccination with a live-attenuated WNV vaccine induced polyfunctional SVG9-specific CD8^+^ T cells in 95% of HLA-A*02:01 positive vaccinated donors, these T cells persisted for a year following vaccination, and SVG9 responsive T cells lysed cells expressing the WNV envelope protein and aided in the control of viral replication [15].

The hypothesis tested here is that, as it is for antibody epitopes, T cells control WNV infections by focusing on particular viral ligands while responses to other viral ligands are less correlative with immune protection. However, the number and nature of viral fragments presented by class I HLA remains an outstanding immunologic question, and the goal of this study was to learn how class I HLA reduce a complex viral proteome to a manageable number of putative immune epitopes and to learn how T cells parse reactivity to the available viral ligands. To test this hypothesis, HLA molecules were gathered from WNV-infected and uninfected cells and their peptide ligands systematically compared [16], [17]. The results reproduce the observation that HLA sample a small number of viral peptides dispersed throughout the flaviviral polyprotein and that T cells focused upon an immunodominant HLA/WNV epitope can be found in patients that have resolved their infection. Given immunodominant WNV T cell epitopes, one can envision the development of immune therapies and diagnostics with T cells directed towards these HLA/WNV epitopes.

## Results

### Six WNV Ligands were presented by Class I HLA-A*11:01

Secreted HLA-A*11:01 class I molecules (sHLA) were gathered from WNV infected and uninfected cells and utilized as a source of endogenously loaded peptides for comparative proteomics. Flow cytometric analysis of the cells producing sHLA confirmed that one set of peptides in the comparative analysis were derived from infected cells because viral particles were detected within sHLA producing cells 12 h after infection of the cells (data not shown). By day 4, 87% of the cells were infected with WNV (Table 1) and A*11:01 containing cell supernatant was harvested from infected cells for days 1 to 5 after infection. sHLA from WNV infected cells were pooled for affinity chromatography purification and infected and uninfected A*11:01 yields were 17 mg and 114.9 mg, respectively, as determined by ELISA.

**Table 1:** sHLA production after introduction of WNV into the bioreactor.

Day	Infection%	sHLA Production (ng/mL)
1	1.31	21.34
2	10.02	16.25
3	61.85	14.27
4	87.21	4.12
5	81.74	NA

**Figure 1:**
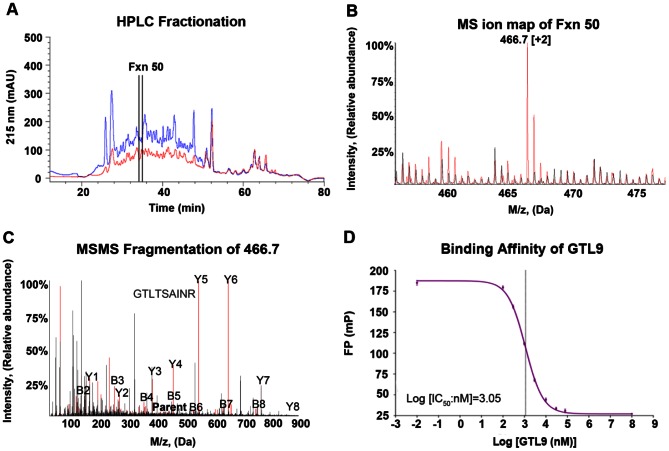
Direct epitope discovery of WNV epitope GTL9. (A) RP-HPLC profile of HLA-A*11:01 peptides from uninfected (blue) and WNV-infected (red) cells. (B) MS ion spectra of HPLC fraction 50 from uninfected (black) and infected (red) cells. MS data were recorded for 300 scans. (C) Overlay of the MS/MS fragmentation pattern of ion 466.7 in the infected HPLC fraction 50 (identified as the GTL9 peptide; red) with the MS/MS fragmentation pattern for the GTL9 synthetic peptide (black). (D) Florescence polarization competitive binding assay with the GTL9 synthetic peptide and HLA-A*11:01.

Peptides eluted from HLA class I molecules contain thousands of different peptide constituents. Prior to comparative mass spectrometric analysis, peptide complexity was reduced by reverse phase HPLC (RP-HPLC) into fractions containing approximately 200–250 peptides each (Figure 1A). Similar HPLC elution patterns for uninfected and infected peptide pools were obtained (Figure 1A). First dimension mass spectrometric (MS1) ion maps showed that the vast majority of ions were shared in the infected and uninfected MS ion maps (Figure 1B). The peptides shared between WNV infected and uninfected cells represent self peptides derived from normal host proteins that are routinely sampled by class I HLA regardless of the cells being infected or uninfected. Figure 1B shows an ion map generated from HPLC fraction 50, where ion 466.7 was identified as unique to the WNV-infected peptide pools. MS2 fragmentation was performed on ions unique to infected cells and individual peptide sequences were determined (Figure 1C). MS2 fragmentation at the same location in the corresponding uninfected fraction (and neighboring uninfected fractions) confirmed the absence of this peptide in uninfected cells. Fragmentation patterns between the newly discovered WNV-derived peptides were compared to the MS2 fragmentation pattern of the corresponding synthetic peptide to ensure amino acid sequence fidelity. Figure 1C shows the MS2 fragmentation pattern of ion 466.7 (identified as WNV-derived GTL9 peptide) overlaid with a synthetic GTL9 peptide demonstrating a correct sequence assignment. Finally, WNV derived peptides were assayed for their binding affinity to HLA-A*11:01.

**Figure 2:**
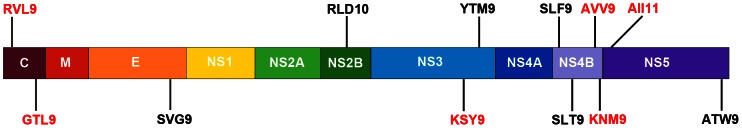
Location of A*11:01 (red) and A*02:01 (black) WNV peptide epitopes on the WNV polyprotein. C, nucleocapsid; M, membrane; E, envelope glycoprotein; NS, nonstructural.

Figure 1D demonstrates that GTL9 is a high-affinity HLA-A*11:01 ligand with an IC_50_ of 1132 nM. Using this direct comparative approach, six HLA-A*11:01 WNV-derived peptides were identified from four different viral proteins (C, NS3, NS4b, and NS5) (Figure 2 and Table 2). Five peptides were nonamers and one peptide was eleven amino acids in length.

**Table 2:** Identified WNV-derived peptides presented by HLA-A*11:01.

Peptide	Sequence	Location	Protein	IC_50_ (nM)
KSY9	KSYETEYPK	1894 – 1902	NS3	7885
AVV9	AVVVNPSVK	2458 – 2466	NS4B	2254
GTL9	GTLTSAINR	89 – 97	C	1132
AII11	AIIEVDRSAAK	2559 – 2569	NS5	11235
RVL9	RVLSLIGLK	23 – 31	C	333.6
KNM9	KNMEKPGLK	2519 – 2527	NS4B	24333.0

These six WNV peptides fit the reported A*11:01-binding motif. Five of the peptides (KSY9, AVV9, GTL9, RVL9 and AII11) had a canonical A*11:01 P2 anchor residue of S, V, T or I; only peptide KNM9 had a noncanonical asparagine at P2. At their C terminus, A*11:01 peptide ligands prefer a lysine anchor, and only GTL9 (C-terminal arginine) did not have a lysine C-terminal anchor. When tested for binding to HLA-A*11:01, these six WNV peptides were found to be high to medium-affinity binders with RVL9 being the strongest binder, having an IC_50_ of 333.6 nM (Table 2). The fluorescence polarization assay used to assess A*11:01 binding categorizes peptides with IC_50_ values <5,000 nM as high affinity. In summary, six WNV ligands were discovered by direct elution from A*11:01, these ligands were sequenced by tandem MS, their sequence was confirmed with synthetic ligands, they bound well to A*11:01, they were of traditional length, and these ligands were consistent with the previously reported A*11:01 motif.

### Class I HLA Sampling of Ligands within the WNV Polyprotein

In addition to learning the number of viral ligands available for immune surveillance, these data indicated the location of class I HLA presented ligands within the WNV polyprotein. In our previous survey of WNV, we found that A*02:01 presents six peptides derived from five different proteins throughout the WNV polyprotein (Figure 2 in black). Here, the HLA-A molecule A*11:01 sampled six peptides derived from 4 viral proteins (Figure 2 in red). Viral proteins NS3, NS4B and NS5 near the C-terminus of the WNV polyprotein provided ligands for both A*11:01 and A*02:01. The capsid viral protein at the N-terminus of the WNV polyprotein provided the immune dominant A*11:01 GTL9 epitope and RVL9; this was distinct as no other HLA class I molecules had been reported to sample a WNV capsid ligand. While a trend emerged whereby N-terminal WNV proteins provide immune dominant epitopes, more HLA than A2 & A11 would need to be studied to confirm this preliminary observation.

### T Cell Recognition of A*11:01 WNV Ligands

In order to measure T cell reactivity to newly discovered WNV ligands presented by the HLA-A*11:01 of the infected cells, CD8^+^ T cells from the peripheral blood mononuclear cells (PBMCs) of twelve A*11:01 WNV-infected patients were stimulated first with a pool of six identified WNV peptides for 9 days, then with each individual peptide for 6 hours, after which T cell responses were tested by intracellular cytokine staining (ICS).

Extended incubation of PBMC with WNV peptides was undertaken for several reasons. First, PBMC were harvested from WNV infected individuals a year or more following the patient’s initial infection. When PBMC stimulated in vitro with specific antigen are assessed for cytokine production, only recently primed T cells tend to be detected [18]. Prolonged incubation of PBMC in the presence of peptides is not uncommon when a subjects’ PBMC are tested long after the peak of an acute infection [19]–[22]. Second, short term incubation of PBMC with peptides tend to reveal dominant viral epitopes while extended cultures are more likely to reveal epitopes that are otherwise undetectable or marginally detectable [23]. Stimulation with the mixture of six WNV peptides was deliberately chosen as the best approximation of the natural setting where infected cells present multiple viral epitopes. It has been demonstrated that this approach identifies a CD8^+^ T cell hierarchy that closely resembles that seen after natural infection [24]. Finally, the exposure of PBMC to a mixture of peptide antigens in a short incubation yielded reactivity only to the most dominant epitope (GTL9) among those discovered here (data not shown). Extended T cell cultures were therefore the best fit for testing dominant and subdominant WNV/HLA epitopes with PBMC gathered approximately 1-year post infection.

**Figure 3:**
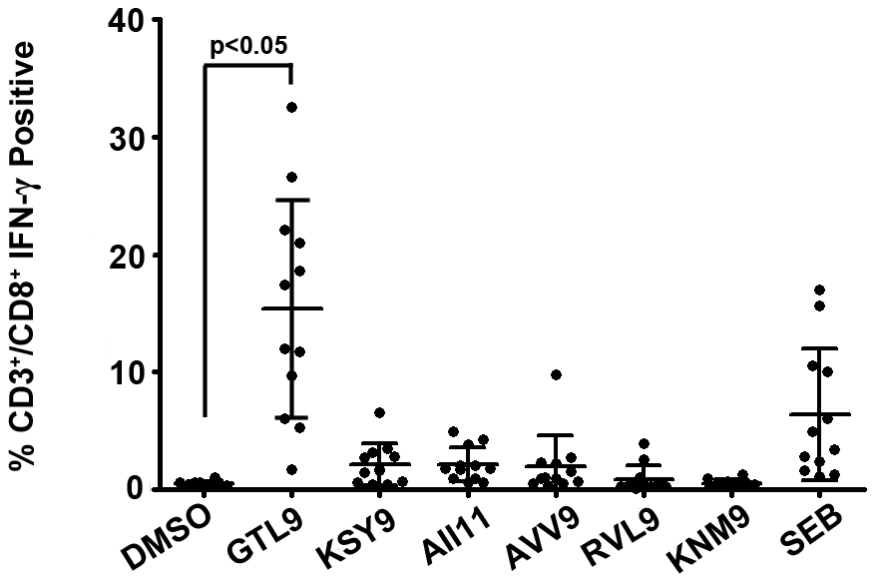
CTL reactivity of identified WNV epitopes. PBMC isolated from naturally infected individuals were stimulated with a pool of 6 WNV peptides in the presence of IL-2 for 9 days. On day 10, cultures were restimulated with individual peptides for 6 h and T cell responses were tested by Intracellular Cytokine Staining. Y-axis  =  %IFN-γ positive CD3^+^/CD8^+^ T cells. Each dot represents the response from a single WNV subject. The average response to GTL9 was significantly higher than the response to the negative control (DMSO). SEB: Staph Enterotoxin B *: significant increase from negative control. Significance was determined by one-way ANOVA followed by Tukey’s test; P<0.05.

**Figure 4:**
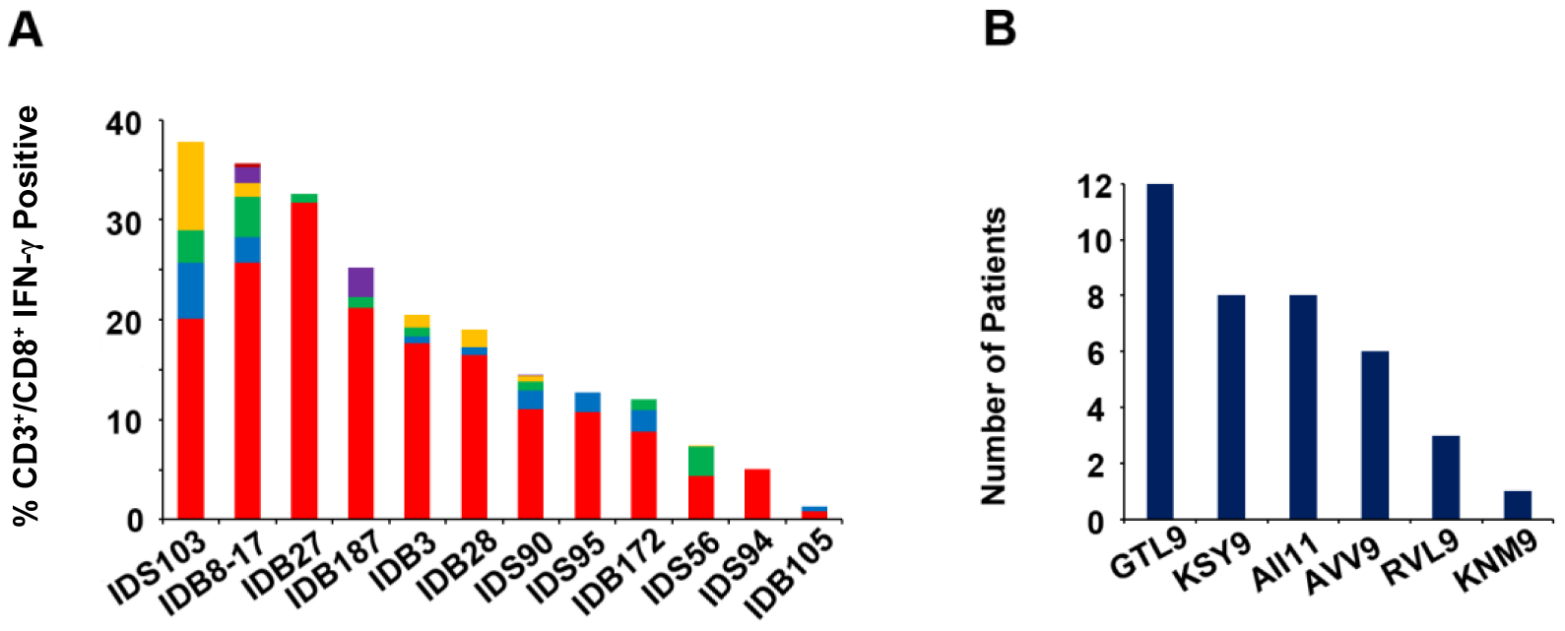
Heterogeneity of CTL responses to WNV. **A.** PBMC isolated from subjects were stimulated with WNV peptides as described in materials and methods and T cell responses were tested by Intracellular Cytokine Staining. Y-axis  =  %IFN-γ positive CD3^+^/CD8^+^ T cells. Each bar represents responses from CD3^+^/CD8^+^ T cells from each subject to the identified WNV peptides. GTL9 (red), KSY9 (blue), AII11 (green), AVV9 (yellow), RVL9 (purple) and KNM9 (brown). Histograms represent patient-specific responses to WNV epitopes minus the negative control (DMSO) plus 2× STD. **B.** Number of patients that recognized each epitope.

In all twelve patients, CD3^+^/CD8^+^ T cell responses predominated to GTL9 [mean: 15.39% of the CD3^+^/CD8^+^ cells were positive for IFN-γ] whereas mean responses to KSY9, AVV9, AII11, RVL9, and KNM9 were 2.12%, 1.94%, 2.10%, 0.88%, and 0.52%, respectively (negative control DMSO mean  =  0.47%) (Figure 3). The WNV peptide epitope KNM9 demonstrated activity above the negative control only in one patient and RVL9 was similarly negative (Figure 4A). These data show that CTL responses to these newly discovered HLA-A*11:01-restricted WNV ligands fall into an immune dominant tier (GTL9), a subdominant tier (KSY9, AVV9, & AII11), and an immunologically inert tier (RVL9 & KNM9). T cell responses to the dominant T cell epitope were comparatively stronger than those to the T cell mitogen SEB because extended incubation with the A11 peptides proceeded for 9 days while stimulation with the SEB (positive control) was performed for 6 hours at day 10.

Individually, T cells from all twelve of the A*11:01 subjects responded to GTL9 (red) as the dominant epitope (Figure 4). As a secondary subdominant epitope, T cells from four subjects recognized KSY9 (blue), 3 recognized AVV9 (yellow), 3 recognized AII11 (green) and 1 recognized RVL (purple) (Figure 4A). As a tertiary epitope, three subjects recognized KSY9, 4 recognized AII11 and 1 recognized AVV9. The CTL response to KSY9, AVV9, and AII11 epitopes were not significantly different from each other among the subjects (Figure 3) and different subjects responded to these 3 epitopes as their second and third epitope at approximately the same frequency. Individuals within this cohort were consistent in their recognition of a dominant WNV epitope yet demonstrated variability in their selection of secondary and tertiary subdominant T cell epitopes.

### T Cell Functional Response to A*11:01 WNV Epitopes

**Figure 5:**
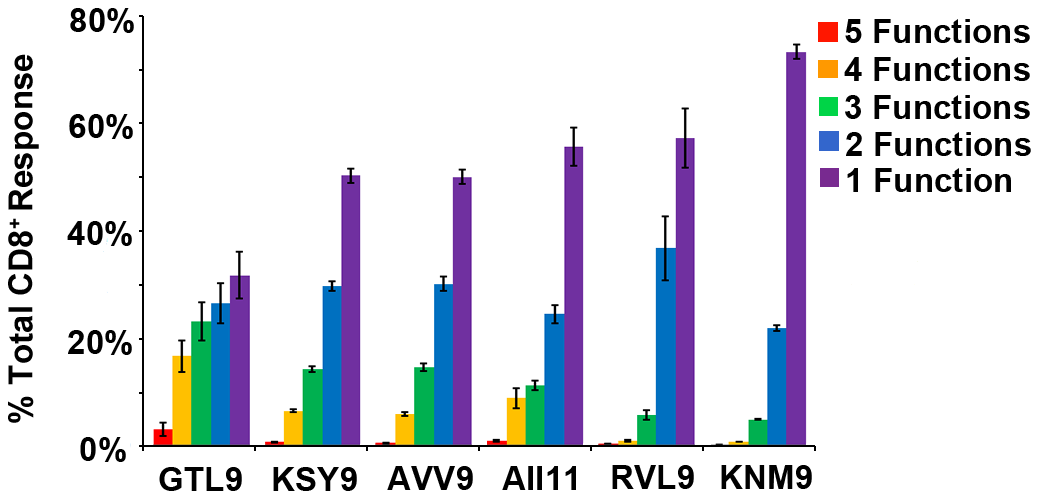
Peptide specific CD8^+^ responses from PBMC isolated from 12 WNV infected subjects stimulated with peptides. Each bar represents the mean percentage of the CD8^+^ T cells from all 12 donors that express 1 (purple), 2 (blue), 3 (green), 4 (yellow) or 5 (red )markers [4 cytokines (IL-2, IFN-γ, TNF-α, and MIP-1β) + degranulation factor (CD107 a)] when exposed to each peptide.

Accumulating data suggest that effective T cell control of flaviviral infections requires anti-viral polyfunctional T cells to secrete multiple cytokines and proliferate more readily on encounter with antigen [25]. Polyfunctional T cells correlate better with protective immunity to viruses than do single cytokine secretors [25], [26]. This includes reports of polyfunctional T cell responses resulting from YFV 17D flaviviral vaccination [27]. To investigate the polyfunctional characteristics of T cells from WNV infected individuals, we stimulated PBMC from twelve A*11:01 infected individuals with the six WNV peptides reported here and analyzed peptide-specific induction of IFN-γ, TNF-α, IL-2, MIP-1β and CD107a (a degranulation marker) in CD8^+^ T lymphocytes (Figure 5).

**Figure 6:**
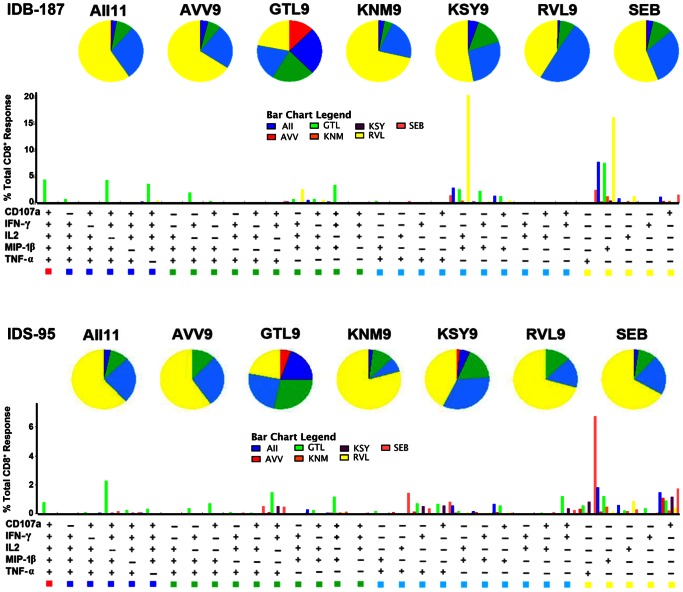
Polyfunctional profiles of WNV-specific CD8^+^ T cells. All possible combinations of five functions (CD107a, IL-2, MIP-1β, IFN- γ, and TNF-α) produced by WNV epitope-specific CD8^+^ T cells are shown on the X-axis. Functional profiles are grouped and color-coded according to number of functions and summarized in the pie charts. Each slice of the pie corresponds to the mean percentage of CD8^+^ T cells producing five (red), four (dark blue), three (green), two (light blue), or one (yellow) function.

**Figure 7:**
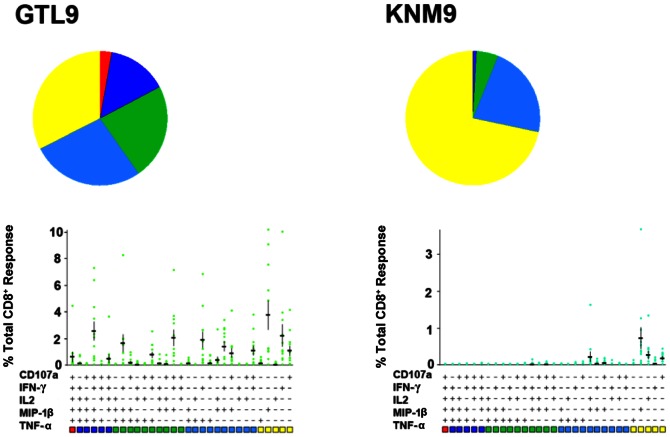
Combined polyfunctional response from 12 individuals to GTL9 and KNM9. PBMC from subjects were stimulated with GTL9 and KNM9 peptide epitopes and functional responses were evaluated by multi-color flow cytometry (as described in materials and methods). Percentages of CD8^+^ T cells expressing the markers shown were input in the SPICE software for display. Each slice of the pie corresponds to the mean percentage of CD8^+^ T cells producing five (red), four (dark blue), three (green), two (light blue), or one (yellow) function. Horizontal bars represent the mean percentage of CD8^+^ T cells producing different possible combinations of the 5 functions in response to the peptides.

Polyfunctional T cells producing ≥4 cytokines were observed in 20% of the total responding cells following stimulation with the immunodominant GTL9 ligand (Figure 5). Subdominant epitopes KSY9, AVV9, and AII11 induced production of 3–4 cytokines in approximately 20% of responding T cells (Figure 5) and ligands RVL9 and KNM9 induced production of 1–2 cytokines in a majority of cells, with only 5% of the responding cells inducing 3 functions (Figure 5). Figure 6 provides polyfunctional T cell responses for two representative individuals in the study, IDB-187 and IDS-095, while figure 7 illustrates the combined polyfunctional response for two representative epitopes, GTL9 and KNM9.

For each individual tested, IFN-γ secreting polyfunctional T cells specific for GTL9 were found at the highest frequency; this was the primary, or immunodominant, response. For the subdominant epitopes KSY9, AVV9, and AII11, no clear epitope hierarchy was found in the patients tested; these lower secondary responses were less polyfunctional with each individual displaying a distinct secondary epitope response hierarchy (Figure 6). In human populations, these data suggest that cellular immunity commits to one epitope, A*11:01 GTL9. Furthermore, the population does not commit to a secondary epitope, such that a WNV GTL9 escape mutant would face different polyfunctional immune pressure on epitopes KSY9, AVV9, and AII11 depending upon the person infected.

## Discussion

Effective anti-viral immune responses tend to be tightly focused on one or a few highly conserved immune epitopes. Factors including epitope accessibility, B cell maturation, and T cell help contribute to the formation of tightly focused neutralizing antibodies [28]–[32]. In regards to cell mediated anti-viral immunity, it is the class I HLA molecules, their intracellular chaperones, and a number of intracellular proteolytic complexes that are responsible for extracting a manageable number of immune epitopes from an entire virus [33]–[38]. Once a ligand or ligands are confirmed as presented by the HLA of virus-infected cells, T cells can proceed to control viral infections via recognition of one or more of the presented epitopes. The purpose of this study was to first understand the number of WNV ligands that are presented by the HLA of infected cells and to then measure T cell reactivity to these ligands. It is our position that a firm understanding of WNV ligand availability and T cell epitope recognition will act as a cornerstone in the development of T cell eliciting vaccines and therapies [39], [40].

The first noteworthy observation of this study is that six WNV derived peptide ligands are presented by HLA-A*11:01 molecules. We previously reported that HLA-A*02:01 presents six WNV ligands, however since A*02:01 peptides are difficult to enumerate by mass spectroscopy because of their non-charged P2 and P9 ligand anchors, in this study we used A*11:01, a class I HLA-A molecule that anchors peptides via an R or K at the C-terminus, making it ideal for mass spectrometric characterization. Interestingly, the A*02:01 results were reproduced with A*11:01. Both HLA-A molecules reduced WNV polyprotein to a small number of available immune ligands. As cellular immune responses are largely effective in containing WNV infections, the HLA-A presentation of a handful of viral ligands can now act as a benchmark of immune success to which other viruses can be compared for class I HLA ligand sampling.

As a segue to the discussion of how T cells manage the six presented WNV ligands, it is worthwhile to consider the location of HLA-A presented ligands within the WNV polyprotein. The immune dominant A*11:01 epitope GTL9 comes from the WNV capsid protein which is the most N-terminal viral protein. By comparison, the dominant A*02:01 epitope SVG9 is derived from the WNV envelope protein, and SVG9 is the most N-terminal A2 ligand detected. The N-terminal nature of these dominant epitopes is consistent with the processing and presentation of defective ribosomal products [41], although the fact that the most N-terminal A*11:01 capsid ligand RVL9 is immunologically inert shows that factors other than ribosome processing contribute to T cell epitope selection. In contrast to the immunodominant GTL9 N-terminal ligand, the subdominant epitopes as well as the immunologically inert ligands are found more towards the C-terminal portion of the virus. These data suggest that immune dominant WNV epitopes are more likely to be derived from the N-terminus of the large flaviviral polyprotein and that less immunogenic ligands will be located further downstream, towards the C-terminus. If dominant viral T cell epitopes are indeed located more towards the N-terminus of the viral polyprotein, inclusion of WNV capsid in addition to matrix, and envelope proteins for WNV vaccine constructs might worth considering. Once we confirmed that a handful of ligands were presented by class I HLA, the breadth and nature of T cell responses to these ligands was tested.

On one end of the spectrum were dominant T cell responses to GTL9. Each infected A*11:01 person dedicated a substantial portion of their cellular immune response to this epitope and these T cells were predominantly polyfunctional. Almost as one, WNV infected A*11:01 positive subjects focused polyfunctional T cells on GTL9. These data parallel the A*02:01 positive subjects response to SVG9 [42]. It has been proposed that polyfunctional T cells, particularly those producing IFN-γ, TNF-α, and IL-2, correlate with protection and control of infectious diseases [19]. This appears to be the case with T cells that recognize the A*02:01 SVG9 epitope, as our previous work suggests that polyfunctional CD8^+^ T cells to SVG9 correspond to the resolution of WNV infections [42]. In a human vaccine trial, SVG9-specific polyfunctional CD8^+^ T cells that produce IFN-γ, TNF-α, and MIP-1β are associated with the control of WNV replication a year following vaccination [15]. Furthermore, Kim et. al. demonstrate that incorporating the SVG9 peptide into a single chain HLA-A2 trimer DNA vaccine induces SVG9-specific CD8^+^ T cells that protect from a lethal WNV challenge and that the adoptive transfer of these T cells protects from infection in the absence of humoral immunity [14]. Immunization of HLA transgenic mice, a human vaccine trial, and adoptive T cell transfer combine to demonstrate that T cells specific for the WNV immune dominant HLA-A2/SVG9 epitope provide protective immunity.

So far, the immunodominant A*11:01 GTL9 epitope characterized here demonstrates a startling similarity to A*02:01 SVG9. The polyfunctional GTL9 specific CD8^+^ T cells tested here were collected from individuals 1-year post infection, suggesting that polyfunctional T cells specific to GTL9, like SVG9-specific T cells, may contribute to protective immunity. While vaccine trials data from animal models and human vaccine studies are not yet available to confirm the role of GTL9-specific T cells in the control of WNV infections, the similarities of A11/GTL9 and A2/SVG9 make such vaccine studies a promising proposition.

In regards to the subdominant A*11:01 epitopes AII11, KSY9, and AVV9, T cell responses varied from person-to-person in regards to hierarchy, or percentage of responding T cells, and monofunctional T cells tended to predominate for these subdominant epitopes. As a population, WNV infected subjects differed in their subdominant epitope hierarchy and in their polyfunctional T cell phenotype as compared to GTL9. Were a WNV GTL9 escape mutant to emerge, these data suggest that the population’s resulting A*11:01 restricted T cell immunity would be evenly split, with some individuals focusing on AII11, some on KSY9, and other A*11:01 individuals focusing on AVV9. Knowledge of these six WNV ligands allows one to visualize the full gradient of T cell responses and provides a foundation for future studies of shifts in T cell immunity that follow viral escape mutations at immune dominant epitopes.

In the United States, by 11 December 2012, cases of WNV infection surpassed that of any other year with 243 deaths, 597 viremic blood donors, and 5,387 cases [2]. It is not clear what factors have led to such a high number of WNV infections in 2012, but this pathogen is now classified as endemic to North America and is likely here to stay due to an ideal combination of birds and mosquitoes [2]. No vaccine or effective treatment is available for WNV, but a number of prophylactic WNV immune therapies are being tested [1]. Now that polyfunctional T cells have been demonstrated to focus on the capsid-derived GTL9 ligand, inclusion of the WNV capsid protein in addition to envelope might worth considering in future YFV/WNV chimeric vaccines to increase T cell immunogenicity and provide higher protection.

In summary, we report the systematic characterization of WNV ligands eluted from the HLA-A*11:01 of virus infected cells. When combined with our previous data for A*02:01, we see that two distinct class I HLA found at high frequency in the population present a small group of viral ligands and that A*02:01 and A*11:01 are able to sample ligands from different viral proteins. Given knowledge of the ligands presented on infected cells, a spectrum of T cell responses then became apparent whereby polyfunctional T cells recognizing a single viral epitope dominated the cellular immune response to WNV. While all T cell responses mediated by all HLA to all viruses may not follow the metric illustrated here, these WNV data provide a benchmark of highly effective WNV HLA antigen presentation and T cell immunity for comparison to other HLA-mediated immune responses for other pathogens. In addition, the A*11:01 data presented here are consistent with a previous study whereby the immunodominant A*02:01 SVG9 envelope epitope acted as a WNV correlate of immunity and led to the successful testing of an SVG9 epitope vaccine [14]. We are therefore optimistic that the immunodomiant A*11:01 capsid-derived GTL9 ligand and the capsid antigen will likewise contribute to next-generation West Nile virus immunotherapies.

## Materials and Methods

### Ethics Statement

Peripheral blood was collected from consenting subjects diagnosed with WNV in heparinized tubes in accordance with a protocol reviewed and approved by the Institutional Review Board at the University of Pittsburgh. For all donors, written, informed consent was obtained before enrollment into the study. The Pittsburgh IRB was prepared in cooperation with the state of Idaho Southwest District Health and the state of Idaho Central District South Health Departments authorizing the University of Pittsburgh IRB to be the IRB of record.

### Virus and Cell Culture

WNV strain WNV NY99 was propagated on Vero E6 cell monolayers [American Type Culture Collection (ATCC) CRL-1586]. Infected cell supernatant was cleared of cell debris by centrifugation at 3,000 × g for 15 min and stored at – 80°C. HeLa (ATCC CCL-2) and Vero cells were subcultured according to ATCC instructions in DMEM F12K (Wisent, Inc.), 10% FBS (Serum Source International), and 1% penicillin/streptomycin (Invitrogen).

### sHLA-Secreting Transfectant Cell Line

HLA-A*11:01 cDNA was amplified by using a reverse oligonucleotide primer that truncated the 3′ end of exon 4, deleting the transmembrane and cytoplasmic domains. Furthermore, the 3′ reverse oligonucleotide primer included a VLDLr purification epitope tag (SVVSTDDDLA) that is recognized by the anti-VLDLr mAb (ATCC CRL-2197). The resulting PCR product was cloned into pcDNA 3.1(–) (Invitrogen) and transfected by Electroporation into HeLa cells. Quantification of sHLA in supernatant was performed by using a sandwich ELISA, where an antibody against the VLDLr epitope was the capture antibody, the primary detector antibody was directed against β_2_-microglobulin (Dako Cytomation).

### Viral Detection

To confirm that HeLa cells were virus-infected, flow cytometry was performed using E24 (Antibody against WNV envelope). WNV infected cells were collected from the bioreactor (CP-2500 Hollow Fiber Bioreactor, Biovest Inc.) fixed in 70% Ethanol and stored at –20°C until required for flow cytometry/FACS. Ethanol was then removed and the cells were resuspended in Cell Staining Buffer (PBS, 1% FBS, 0.01% NaN3) to allow rehydration. The cells were pelleted again and incubated with AF 647-Conjugated E24 (kindly provided by Dr. Michael Diamond, Washington University, St. Louis, MO) at 4°C for 30 minutes. The cells were then washed and resuspended in Cell Staining Buffer and transferred to FACS tubes. Flow cytometry was performed using a FACSCalibur flow cytometer (Becton Dickinson, Mountain View, CA). Data files were acquired using BD CELLQuest 3.3 software and analyzed by flowJo V 7.8. (TreeStar Inc.).

### Production and Isolation of HLA-A*11:01 Peptides

sHLA-A*11:01 peptide complexes were produced in a bioreactor format as described [17], [43], [44]. To obtain sHLA from WNV-infected cells, bioreactors containing HeLa cells were infected with 2×10^8^ pfu in a total volume of 200 ml (MOI ≈ 3×10^−4^) and recirculated in the bioreactor for 2 h before the harvest of sHLA-containing supernatant was initiated. Supernatant from days 0.5 to 5 after infection were pooled for purification because we needed 12 h to flush uninfected supernatant from the system. sHLA complexes were purified by affinity chromatography with the anti-VLDLr epitope antibody. Acid eluted peptides were lyophilized and then fractionated by RP-HPLC with a Jupiter Proteo 90 Å, 4 µm, 150×2 column (Phenomenex) [16].

### MS Analysis and Peptide-Binding Assay

MS ion maps were generated for HPLC fractions containing peptides. Fractions were ionized by using nanoelectrospray capillaries (Proxeon) into a QSTAR elite (Applied Biosystems) electrospray quadrupole time-of-flight mass spectrometer. Peptide sequences were determined manually by using the MASCOT software package (Matrix Science). Peptide binding to HLA-A*11:01 was determined using the florescence polarization method (Pure Protein L.L.C.) [45].

### WNV Patients and Samples

For this study PBMC were collected from 129 WNV infected patients, recruited between March and June 2007 from high-incidence areas in the central and southwest health districts of the State of Idaho. The median age of cohort was 51 years, and the range was 24–71 years. All persons had been diagnosed with WNV in the summer of 2006, with confirmatory testing conducted by the Idaho State Health Department. After collection, blood samples were shipped overnight to Pittsburgh where PBMC were isolated on Ficoll–Hypaque density gradients and cryopreserved in 10% DMSO. Hemolyzed blood samples or samples that had been in transit for over 24 hours after collection were discarded. The WNV serologic status of the Idaho cohort was further confirmed by IgM/IgG ELISA testing by the Virology & Immunology Section Pennsylvanya Department of Health Bureau of Laboratories.

HLA typing was completed using high resolution DNA sequenced based typing in an ASHI/CLIA accredited clinical HLA laboratory at the University of Oklahoma Health Sciences Center and 12 patients were identified as A*11:01 positive of which eleven had WNV fever without severe symptoms (non-neuroinvasive disease) and one (IDS-056) had neuro-invasive disease (encephalitis). This cohort was all North American and A*11:01 allele frequency for North American Asian, Hispanic, African American, and Caucasian peoples is 17.1%, 4.3%, 1.1% and 6.3% respectively [46].

### Extended T Cell Cultures

PBMC were thawed, allowed to recover for two hours at 37°C, and then incubated for 9 days in the presence of a pool of six A*11:01 WNV peptides (individual peptide concentration 1 µg/ml) in complete medium (RPMI-1640; 10% heat inactivated fetal calf serum, GemCell; 10 U/ml Penicillin and 10 mg/ml Streptomycin; 25 nM HEPES). Interleukin 2 (500 U/ml, Prometheus Laboratories Inc.) was added on day 3 and then again on day 7. Cultures were washed twice to remove antigen and IL-2 on day 9 and rested in complete medium for 24 hours before performing Intracellular Cytokine Staining (ICS).

### Intracellular Cytokine Staining (ICS)

The polyfunctionality of stimulated T cells was determined by intracellular cytokine staining (ICS) and flow cytometry. At the end of the extended PBMC cultures, cells were incubated for 6 hours with individual peptides (10 µg/ml) or DMSO (as the negative control) in the presence of extracellular transport inhibitors (GolgiStop - GolgiPlug, BD). At the end of the incubation period, cells were stained with the amine-binding dye Aqua (Invitrogen) to identify and exclude of dead cells. Cells were then surface stained with monoclonal antibodies to assign T cell lineage (CD3-APC-H7; CD8-PerCP-Cy5.5; CD4-V450), and after permeabilization with FACS Permeabilizing Solution 2 (BD), T cell function (CD107a-FITC; MIP-1β-PE; IL-2-APC, TNFα-PE-Cy7; IFN-γ-AF700). All monoclonal antibodies were from BD Bioscience. Events were acquired with a LSR-II 12-color flow cytometer (BD Biosciences) and then analyzed using FlowJo V 7.8 (TreeStar Inc.). The cytometer performance and optimal PMT voltages for individual channels were assessed by running CST beads (BD) following the manufacturer’s recommendations. The number of CD3^+^/CD8^+^ events gated for each stimulation was at least 10^5^ (Figure S1 for gating strategy). Positive gates for each of the five functions (Figure S2) were drawn based on the negative control for each fluorescence channel (Figure S3). Boolean gate arrays were created in FlowJo to determine the percentages of the 32 possible combinations (2^5^) of the five functions we examined. For each of the polyfunctional combinations, nonspecific background from the negative control was subtracted from the one obtained from cultures incubated with peptide stimuli and displayed using SPICE (Simplified Presentation of Incredibly Complex Evaluations, Version 5.2, Mario Roederer, Vaccine Research Center, NIAID, NIH).

### Statistical Analysis

Significance was determined by one-way ANOVA followed by Tukey’s method. A *P* value of <0.05 was considered significant. Statistical analysis was performed by using GraphPad Prism 5.0 software (GraphPad Software, Inc.)

## Supporting Information

Figure S1 **Flow cytometry gating strategy.** Cells were stimulated with the appropriate antigenic stimulus and stained as detailed in the materials and methods section. Forward and side scatters were used to gate in the lymphocyte population. This was followed by exclusion of doublets by forward scatter area versus height. Live cells only were then gated in by excluding events that were positive for the amine binding dye excited by the violet laser. CD3^+^/CD8^+^ double positive cells were gated in followed by enumeration of positive cells for each immune functions (only IFN-γ is shown). Representative gating strategy for subject IDB-003 is shown.
(TIF)

Figure S2 **Functional responses to WNV epitope stimulation.** The dot plots show the gated CD3^+^/CD8^+^ events for each of the five functions studied. Gates for each function were positioned based on the negative control (DMSO). Representative dot plots for subject IDB-003 are shown.
(TIF)

Figure S3 **Flow cytometric analysis of CTL IFN-γ responses to WNV epitopes.** Cultures were stimulated for 6 hours in the presence of the indicated stimuli and negative control (DMSO). Cells were challenged with the individual peptide epitopes after extended incubation with pools of WNV peptide (as described in materials and methods). Percentages shown in each dot plot indicate the magnitude of the response to each stimulus as measured by IFN-γ production by CD3^+^/CD8^+^ double positive cells. Representative experiment for subject IDB-003 is shown.
(TIF)
