# Supplementary figures and images for: Identification of Class I HLA T Cell Control Epitopes for West Nile Virus
Source: PLoS One. 2013 Jun 10;8(6):e66298. doi: 10.1371/journal.pone.0066298 (PMC3677933; doi:10.1371/journal.pone.0066298)

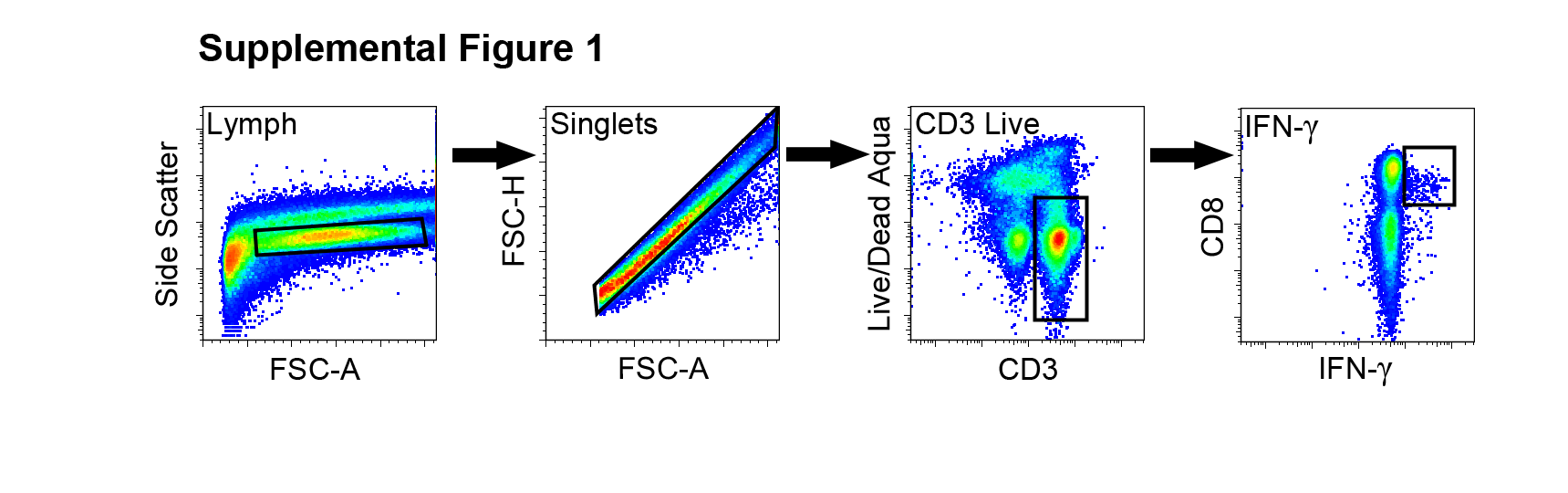

Supplement: Figure S1 — Flow cytometry gating strategy. Cells were stimulated with the appropriate antigenic stimulus and stained as detailed in the materials and methods section. Forward and side scatters were used to gate in the lymphocyte population. This was followed by exclusion of doublets by forward scatter area versus height. Live cells only were then gated in by excluding events that were positive for the amine binding dye excited by the violet laser. CD3+/CD8+ double positive cells were gated in followed by enumeration of positive cells for each immune functions (only IFN-γ is shown). Representative gating strategy for subject IDB-003 is shown. (TIF) [file pone.0066298.s001.tif]

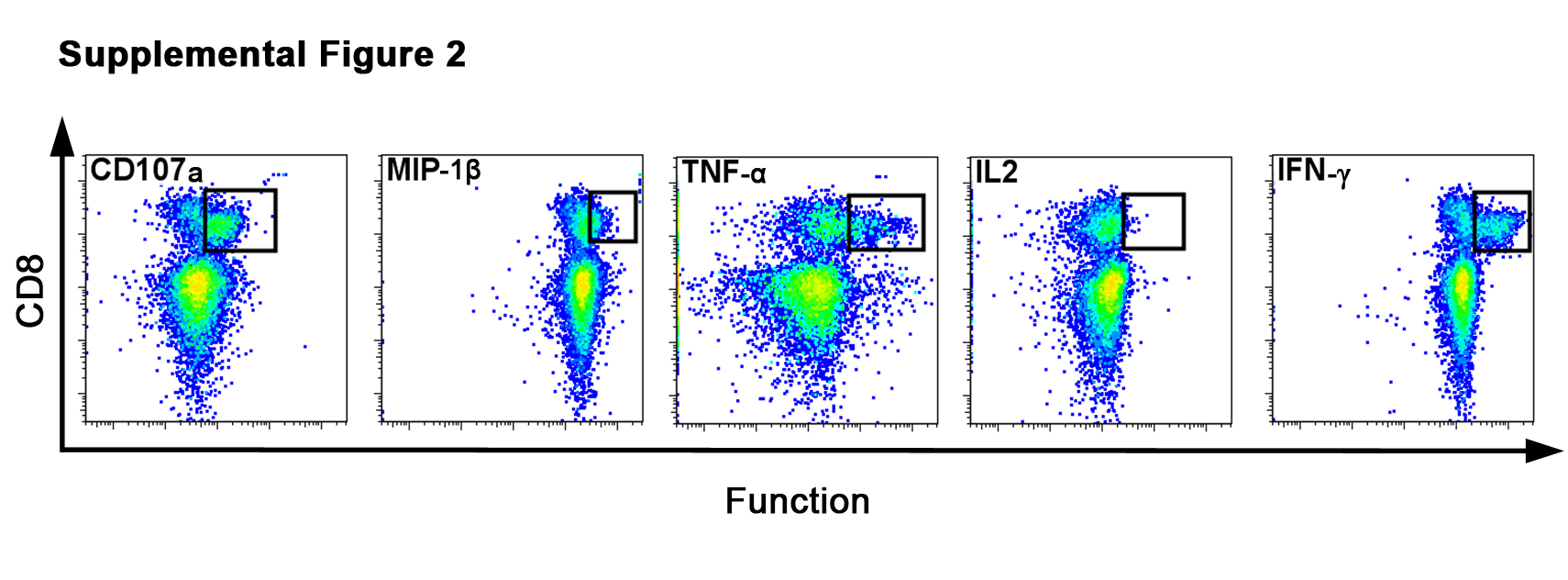

Supplement: Figure S2 — Functional responses to WNV epitope stimulation. The dot plots show the gated CD3+/CD8+ events for each of the five functions studied. Gates for each function were positioned based on the negative control (DMSO). Representative dot plots for subject IDB-003 are shown. (TIF) [file pone.0066298.s002.tif]

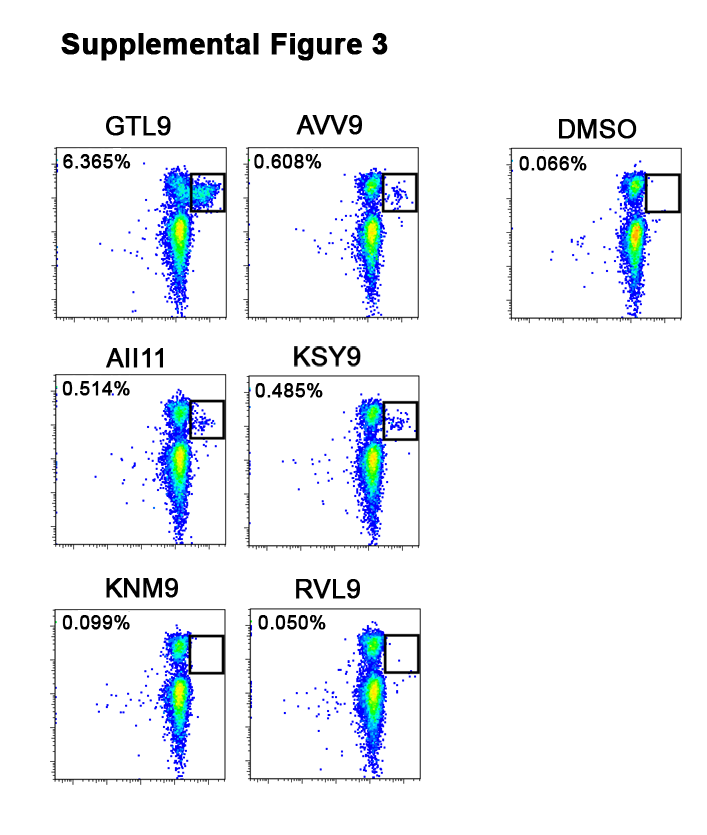

Supplement: Figure S3 — Flow cytometric analysis of CTL IFN-γ responses to WNV epitopes. Cultures were stimulated for 6 hours in the presence of the indicated stimuli and negative control (DMSO). Cells were challenged with the individual peptide epitopes after extended incubation with pools of WNV peptide (as described in materials and methods). Percentages shown in each dot plot indicate the magnitude of the response to each stimulus as measured by IFN-γ production by CD3+/CD8+ double positive cells. Representative experiment for subject IDB-003 is shown. (TIF) [file pone.0066298.s003.tif]
